# Experimental Generation of SNP Haplotype Signatures in Patients with Sickle Cell Anaemia

**DOI:** 10.1371/journal.pone.0013004

**Published:** 2010-09-24

**Authors:** Stephan Menzel, Jian Qin, Nisha Vasavda, Swee Lay Thein, Ramesh Ramakrishnan

**Affiliations:** 1 Division of Gene and Cell Based Therapy, King's College London School of Medicine, London, United Kingdom; 2 Fluidigm Corporation, South San Francisco, California, United States of America; 3 Department of Haematological Medicine, King's College Hospital, London, United Kingdom; Instituto de Biomedicina de Valencia, CSIC, Spain

## Abstract

**Background:**

Sickle cell anemia is caused by a single type of mutation, a homozygous A→T substitution in the ß globin gene. Clinical severity is diverse, partially due to additional, disease-modifying genetic factors. We are studying one such modifier locus, *HMIP* (*HBS1L*-*MYB* intergenic polymorphism, chromosome 6q23.3). Working with a genetically admixed patient population, we have encountered the necessity to generate haplotype signatures of genetic markers to label genomic fragments with distinct genealogical origin at this locus. With the goal to generate haplotype signatures from patients experimentally, we have investigated the suitability of an existing nanofluidic assay platform to perform phase alignment with single-nucleotide polymorphism alleles.

**Methodology/Principal Findings:**

Patient DNA samples were loaded onto Fluidigm Digital Arrays and individual DNA molecules were assayed with allele-specific probes for SNP markers. Here we present data showing the utility of the nanofluidic approach, yielding haplotype data identical to those obtained with a family-based method. We then determined haplotype composition in a group of patients with sickle cell disease, including in those where a mathematical inference approach gave ambiguous or misleading results. Experimental phasing of genotypes across 3.8 kb for rs9399137, rs9402685, and rs11759553 created unequivocal haplotype signatures for each of the patients. In 68 patients, we found 8 copies of a haplotype signature (‘C-C-T’), which is known to be prevalent in Europeans but to be absent in West African populations. We have confirmed the identity of our phased allele pairs by single-molecule sequencing and have demonstrated, in principle, that three-allele phasing (using three colors) is a potential extension to this method.

**Conclusions/Significance:**

Phased haplotypes yield more information than the individual marker genotypes. Procedures such as the one described here would therefore benefit genetic mapping and functional studies as well as diagnostic procedures where the identity or parental origin of short genetic fragments is of importance.

## Introduction

Sickle cell anemia is a devastating autosomal-recessive genetic condition [MIM 603903], caused by a mutation in the gene (*HBB*, on chromosome 11p15.4) coding the beta hemoglobin polypeptide, which provides two of the four subunits of adult hemoglobin. While the homozygous mutant genotype is the same in all patients, other genetic factors contribute to marked clinical variability. Several modifier loci act through promoting the persistence of fetal hemoglobin (HbF) into adulthood, which can stand in for the mutated adult hemoglobin and lead to a milder disease phenotype. One such locus is the *HBS1L*-*MYB* intergenic polymorphism, block 2 (*HMIP-2*) [1] on chromosome 6q23.3.

Our patients are of Caribbean and West African origin, but the presence of European genetic admixture provides for additional genetic complexity. To address the confounding influence of non-African alleles in our patients, we are conducting a survey of haplotypes at HMIP locus. Haplotypes are arrays of marker alleles on each of the two sister chromosomes at a given genetic locus. Traditionally they are detected through family studies or by mathematically inferring them from marker genotypes. Since family members for our patients are not available to study, and mathematical inference is occasionally not conclusive, it was our goal to devise a novel, direct lab-based method to align marker alleles into haplotypes, using a nanofluidic approach.

**Figure 1:**
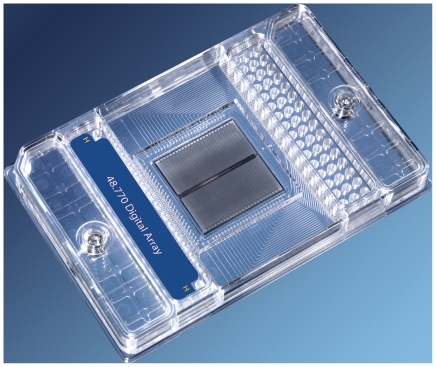
48-panel nanofluidic array. 2^nd^ generation Fluidigm Digital Arrays™ provide for 48 independent haplotyping experiments with each 2-h run of the platform. Loading wells for the 48 experiments are at the top end of the array. Each well leads to a panel of 770 reaction chambers, in which individual target molecules can be isolated and assayed.

**Figure 2:**
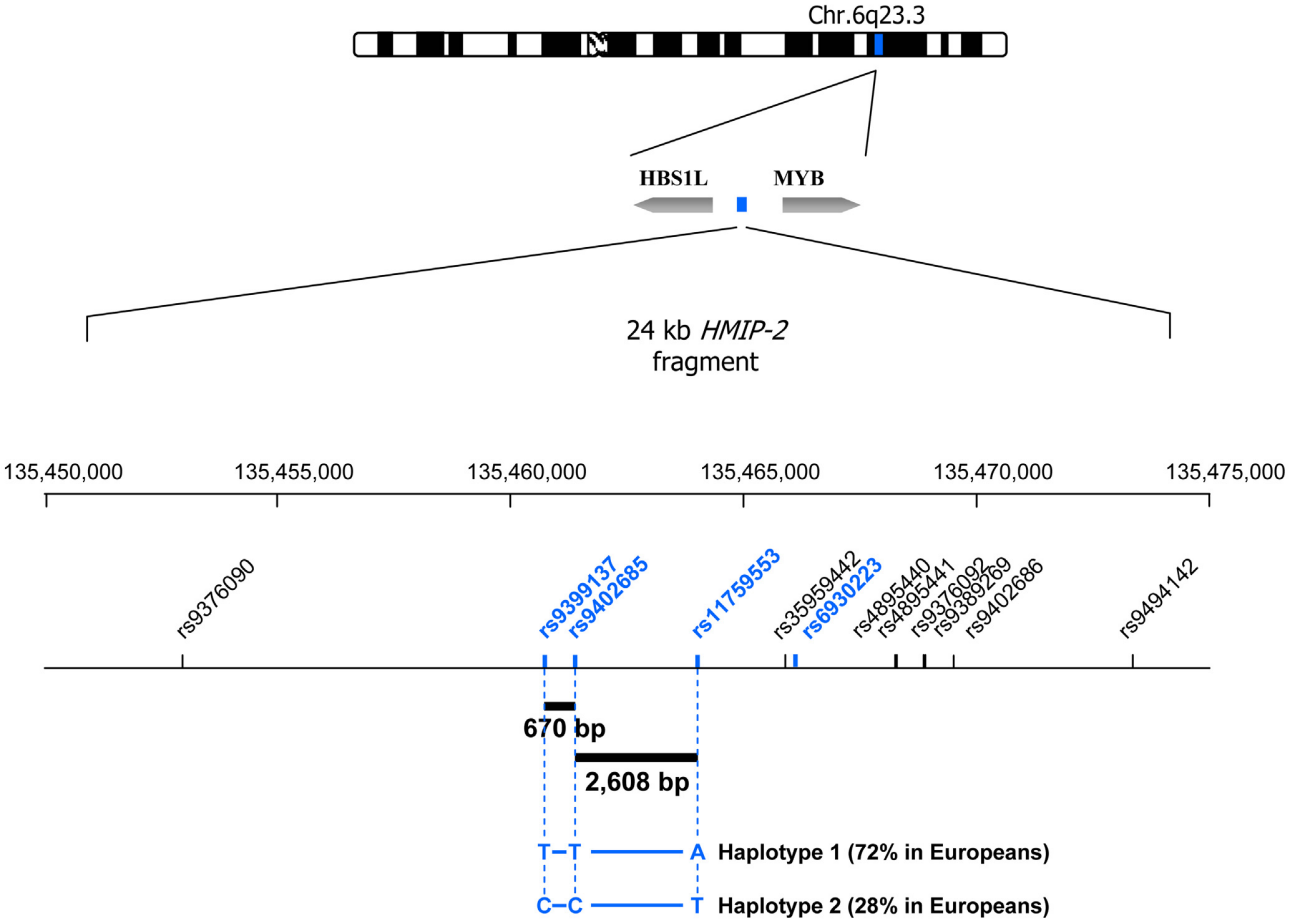
The *HMIP-2* region on chromosome 6q. The locus consists of array of intergenic SNPs on human chromosome 6q23.3, termed *HMIP-2* (*HBS1L-MYB* intergenic polymorphism, block 2). In Caucasian populations, SNP alleles are locked into two frequent haplotypes, which underlie variability in the level of residual fetal hemoglobin (HbF) in adult red blood cells. The causative sequence variant contained here is so far unknown. Haplotype 2, identified here by the short haplotype signature ‘C - C - T’, promotes HbF persistence [1] and can alleviate sickle cell disease and β thalassaemia. In Figure 3, this haplotype is represented by ‘C - C’. To experimentally detect these haplotypes in unrelated individuals, phasing experiments have to bridge a distance of 670 bp between rs9399137 and rs9402685, and a distance of 2,608 bp between rs9402685 and rs11759553. rs35959442 was previously cited as rs52090909 and rs9494142 as rs11154792. Locations and distances are from UCSC Genome Browser, v. March 2006, the ideogram, with permission, from Michelle M Le Beau, Chicago.

It has been shown previously that experimental haplotyping in individual samples can be achieved [2], [3], [4], [5], [6], [7], [8], but it is the advent of high-throughput ‘digital’ technologies [9], [10], [11], [12], [13], [14] that probe individual sample DNA molecules, which offers efficient approaches to phase-defined genotyping that would be amenable to the large-scale studies of complex genetic conditions. We have used one such newly-established high-throughput sequence detection platform using ‘digital’ assaying, the Fluidigm Digital Array™ (Figure 1). The Digital Array platform uses nanofluidics to radically partition DNA samples on a chip, to a level where individual target molecules are isolated and can be individually probed. Thus, target strands derived from maternal or paternal chromosomes can be genotyped separately for neighboring SNPs (single-nucleotide polymorphisms), and phase/haplotype information for these SNPs can be directly derived. We selected three SNPs within *HMIP-2*, including the tag SNP for the locus, rs9399137, as well as two neighboring SNPs, rs9402685 and rs11759553 (Figure 2). Separate phasing experiments were conducted in double-heterozygous patients for the marker pairing rs9399137 and rs9402685, 670 bp apart, and the pairing rs9402685 and rs11759553, 2,608 bp apart.

Here, we first describe a procedure to use Fluidigm Digital Arrays for the rapid generation of phase information from genotypes, we then show that haplotypes generated in such way are accurate, i.e. identical to those generated by phase assignment within a family and finally, we investigate a group of sickle cell patients to identify distinct haplotypes in each individual through three-SNP haplotype signatures.

## Methods

### Subjects

**Figure 3:**
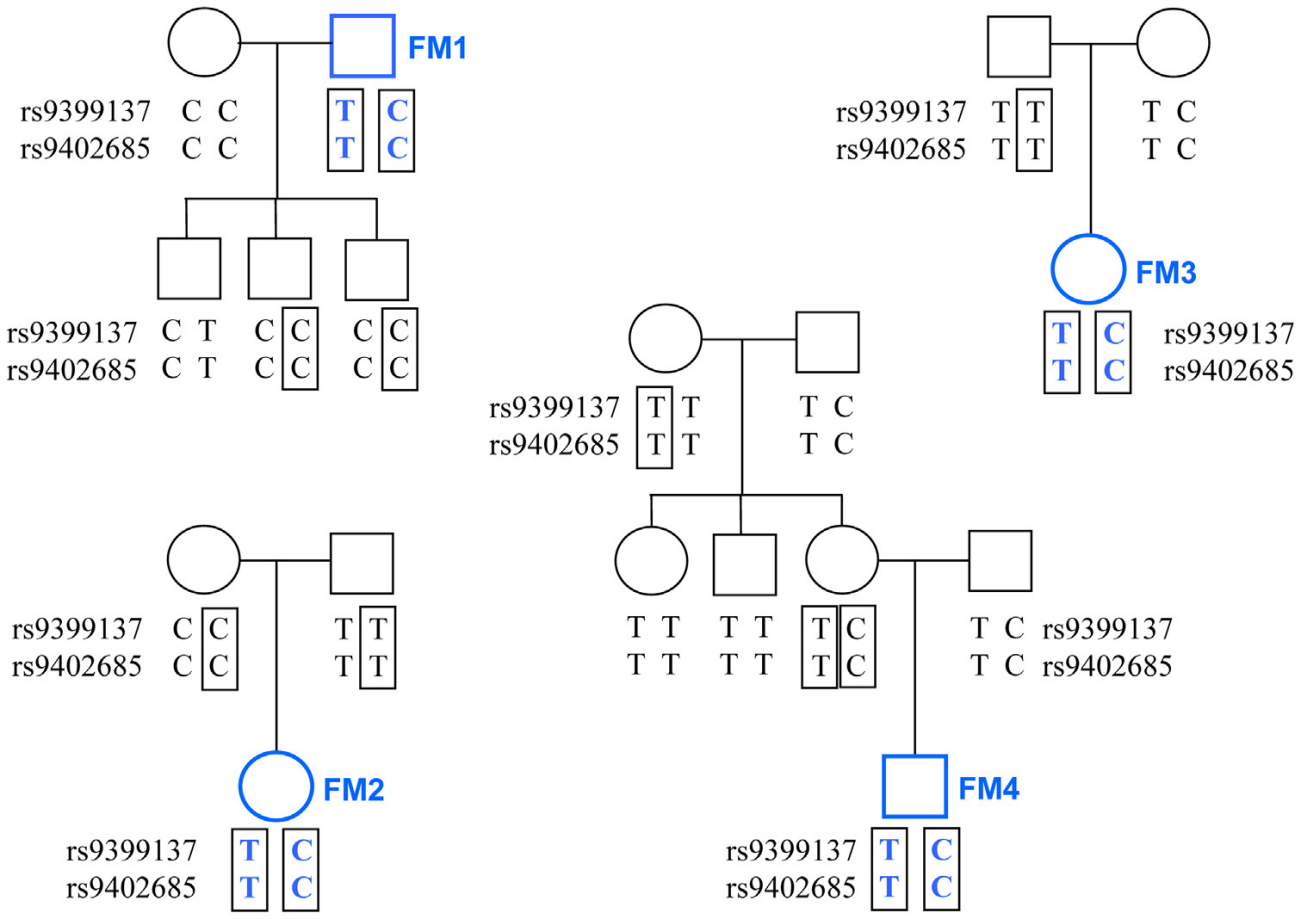
Family-based haplotyping in individuals FM 1, 2, 3 and 4. All individuals are part of the same extended family, and the complete haplotypes (not shown) contain >80 SNP markers and microsatellites. Boxes illustrate how phase is derived initially from homozygous individuals, and then propagated through the pedigree sketch. As typical for European and South Asian Caucasian individuals, only two of the four possible haplotypes occur. In this family, haplotype ‘C – C’ is associated with raised HbF and milder thalassemia [1], [15].

Four members (Figure 3) of a large family, which originates from North West India and which segregates β thalassaemia and heterocellular hereditary persistence of fetal hemoglobin, have previously been studied to investigate the influence of the *HMIP* loci [15].

68 patients with sickle cell anemia (HbS homozygous) are part of a study to investigate the effects of *HMIP-2* [16] and other loci on fetal hemoglobin levels and disease severity. Patients had complete genotype data for SNPs rs9399137, rs9402685, and rs11759553. Patients are of West African and Afro-Caribbean ancestry with European genetic admixture. SNPs genotyped in these patients show distinct linkage disequilibrium (LD) relationships in European and African descended populations [16]. In Europeans, LD is very tight, with D′ = 1/r^2^ = 1 for rs9399137–rs9402685 and D′ = 1/r^2^ = 0.88 for rs9402685–rs11759553. In an admixed Caribbean population, LD was found to be weaker, with D′ = 1/r^2^ = 0.11 for the first pairing and D′ = 0.49/r^2^ = 0.16 for the second [16]. Phase information for theses SNPs is intended to ultimately aid fine-mapping of this region in patients with sickle cell disease.

The software package PHASE v2.1.1 [17] was used to mathematically infer haplotypes from genotypes alone, prior to obtaining experimental phase information.

Ethical approval has been given for our ongoing studies to investigate HMIP-2 and other disease modifier loci (King's College Hospital Local Ethics Research Committee, LREC 01-083). Written informed consent was given by all subjects.

### Experimental haplotyping procedure

The ***Fluidigm Digital Array*** [18], [19], [20], [21], [22] is a nanofluidic biochip where digital PCR reactions can be performed with isolated individual DNA template molecules. It is conventionally used for absolute quantitation (“molecule counting”) of genomic DNA and cDNA, rare mutation detection [18], and determination of copy number variation [20], [21] (CNV). Utilizing soft lithography and silicone rubber to create nanoscale valves and pumps, the digital array delivers mixtures of sample and PCR reagents into individual reaction chambers. We have utilized both 1^st^-generation digital arrays [18], [19], [20], [21], [22], which partition 12 samples into 765 reaction chambers each, as well as a 2^nd^ generation digital arrays, which are capable of dividing up to 48 different samples into 770 reaction chambers each. We have previously [20] shown that one can use the digital array for multiplex PCR, using TaqMan assays with different fluorophores, thereby identifying multiple loci in a single reaction chamber.

A PCR/probe reaction mix (for two neighboring SNPs) contained 1× TaqMan gene expression master mix (Applied Biosystems, Foster City, CA), 900 nM each of the four primers, 200 nM each of the two probes (FAM and VIC-labeled, respectively, detecting one allele each of both SNPs), and subject DNA at appropriate concentration so that there will be 70–100 genomic DNA molecules in each of the panels.

Primers and allele probes used here were designed at the Centre National de Génotypage, Evry, France, and are the same as were used in conventional TaqMan genotyping assays (Applied Biosystems, Foster City, CA) in our previous studies [1] (for all oligonucleotide sequences, see Methods S1). All primers were supplied by Integrated DNA Technologies (Coralville, IA) and TaqMan MGB (minor-groove binding) probes containing fluorophores and non-fluorescent quenchers were from Applied Biosystems (Foster City, CA). After sample loading, the digital array was thermocycled on the BioMark system (http://www.fluidigm.com/products/biomark-main.html), which included a 95°C, 10-minute hot start followed by 50 cycles of two-step PCR: 15 seconds at 95°C for denaturing and 1 minute at 60°C for annealing and extension. FAM and VIC signals were recorded at the end of each PCR cycle and the Digital PCR Analysis software (Fluidigm, South San Francisco, CA) was used to process the data after the reaction.

Chambers that yielded signals for either probe alone or for both probes were detected and counted. Co-localization was calculated as follows: in each panel there are *m* FAM-positive chambers and *n* VIC-positive chambers. *m* and *n* are close but not necessarily the same due to DNA breakage and random sampling. The number of chambers positive for both FAM and VIC is *p*. “Co-localization index” would be expressed as *p* divided by the smaller of *m* or *n*.

### Single-molecule sequencing

This method was used as an additional check on our digital-array haplotype alignments.

DNA samples from seven patients (those listed in Table S1) and from family member FM01 were diluted to single molecule level and amplified across rs9399137 and rs9402685 on an ABI 7900 real time PCR instrument (Applied Biosystems, Foster City, CA). Forty 10-µl reactions were performed for each sample, containing 1× TaqMan gene expression master mix (Applied Biosystems, Foster City, CA), 900 nM each of the primers C6-F and C6-R, 0.2× Sybr Green (Invitrogen, Carlsbad, CA) and DNA molecules. Cycles were 95°C, 10 minutes and 50 cycles of 15 seconds at 95°C and 1 minute at 60°C. Efficiency was monitored by a melting curve step. 85 PCR products were sequenced by Sequetech (Mountain View, CA) with both primers, C6-F and C6-R. 74 of these PCR were apparently from single molecules because they showed unequivocal sequencing traces and singular base calls in both SNP positions.

## Results

### A procedure for rapid haplotyping with digital arrays

DNA samples are chosen form individuals that are double-heterozygous for the SNP pair to be haplotyped. Genomic DNA is mixed with PCR reagents for both SNPs, including primers and probes, and loaded onto ‘panels’ of reaction chambers, where it is partitioned so that individual target strands can be assayed in TaqMan reactions with one probe per SNP.

**Figure 4:**
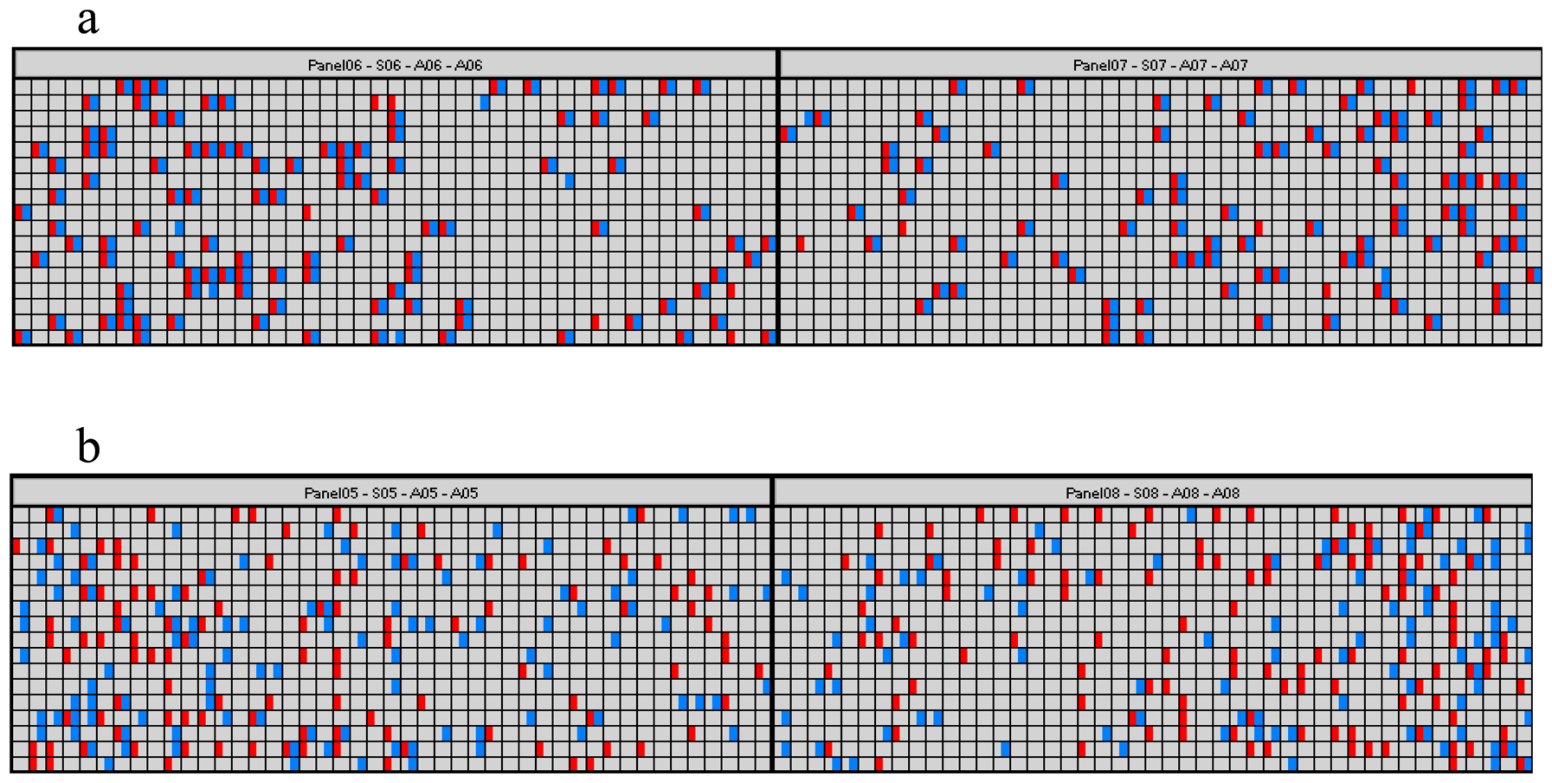
Digital-array haplotyping of SNP pair rs9399137–rs9402685. Shown is the graphical representation of the signal distribution on four panels of a digital array by the Digital PCR Analysis software. (a) Two samples phased using probes for rs9399137-**T**-FAM with rs9402685-**T**-VIC, resulting in a co-localization index of 95% and 98%, (b) the same two samples phased with rs9399137-**T**-FAM and rs9402685-**C**-VIC, resulting in a co-localization index of 19% and 15%.

As an example, the dispersion of 459 pg of sample DNA (containing 70 copies of a SNP allele from a heterozygous genotype) across the 765 chambers of a panel results in most chambers being unoccupied with target DNA sequence, but those 70 copies present are mostly isolated in individual reaction chambers. Each occupied chamber generates a fluorescent signal for the allelic probe used in the PCR reaction. A second allele, belonging to a neighboring SNP and assayed with a differently-labeled probe, is present in the same set of chambers only if it is located on the same DNA double-strand molecule, i.e. ‘in phase’ with the first allele (Figure 4a). If the second allele sits on the other sister chromosome, i.e. is ‘in repulsion’ to the first allele, an entirely independent set of 70 chambers generates a different fluorescent signal for this probe (Figure 4b). Under this second scenario, the two probes may ‘co-localize’ only stochastically, i.e. in a small number of chambers (for this example 765×0.09^2^, i.e. 6 to 7 in each panel).

After thermocycling and real-time fluorescence detection, data are analyzed using the Digital PCR Analysis software (Fluidigm, South San Francisco, CA), which detects and counts co-localizing and isolated probe signals. Results are scored as ‘in phase’ (predominant co-localization) or ‘in repulsion’ (predominant isolation of signals).

**Figure 5:**
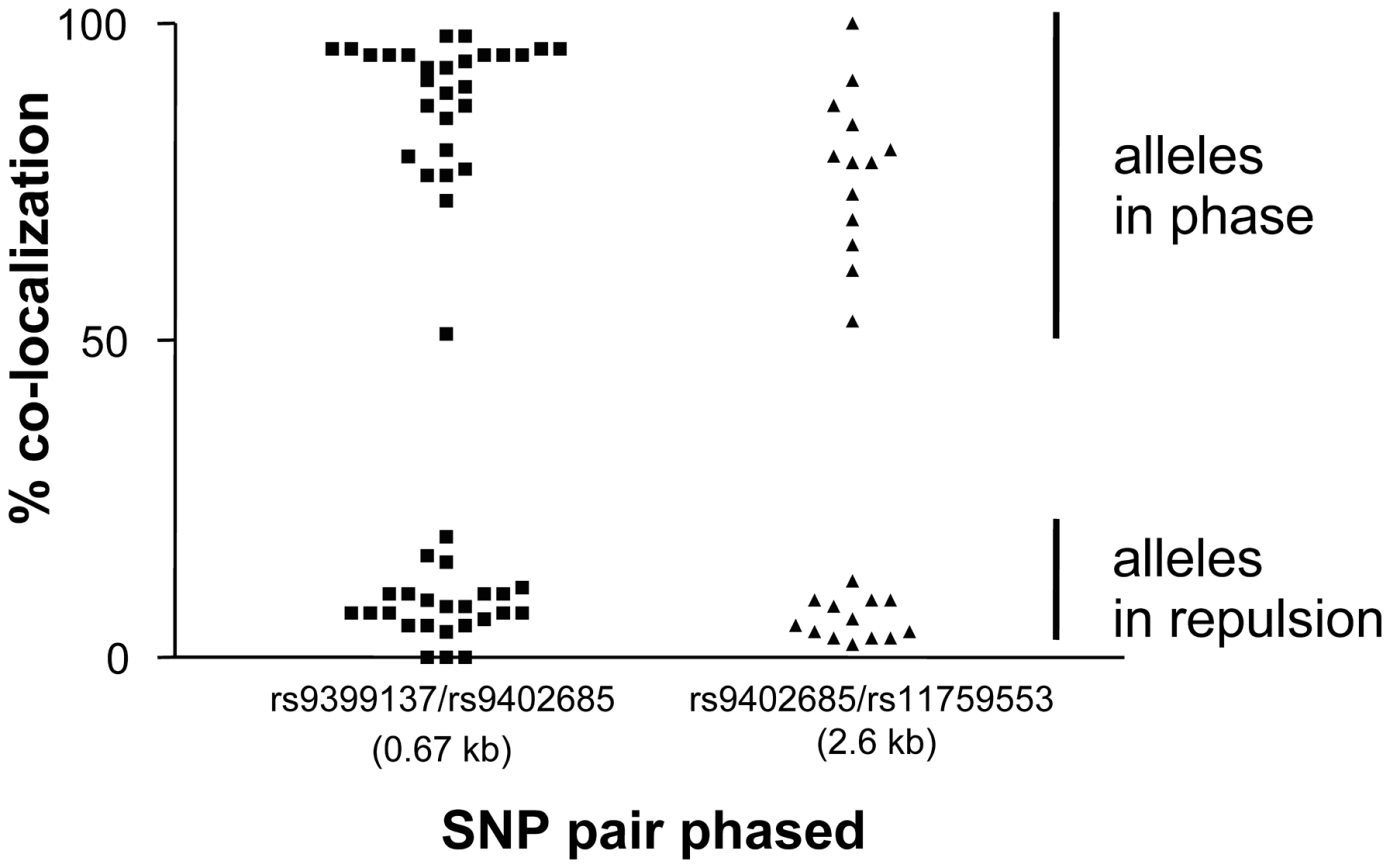
Co-localization index results for both primer pairs tested. This graph represents a summary of all digital-array haplotyping experiments carried out. Notable is the very distinct clustering for alleles in phase and those in repulsion.

While the actual co-localization data were subject to some experimental variability, they grouped, across all our results, into two completely separated clusters (Figure 5, Table 1, Tables S1 and S2). Co-localization indices between 50% and 100% were seen for alleles in phase, and <20% for alleles in repulsion.

**Table 1:** Digital-array haplotyping: Test for accuracy of the approach in a family study.

	FAM allele	FAM targets detected	VIC allele	VIC targets detected	Chambers with both signals	Chambers with either signal	Minimum of the two (FAM or VIC)	% co-localization	Haplotypes derived
**FM1**	rs9399137-T	120	rs9402685-T	173	112	181	120	93% (in phase)	T-T and C-C
	rs9399137-T	124	rs9402685-T	163	111	176	124	90% (in phase)	T-T and C-C
	rs9399137-T	147	rs9402685-C	149	23	273	147	16% (in repulsion)	T-T and C-C
	rs9399137-T	144	rs9402685-C	133	13	264	133	10% (in repulsion)	T-T and C-C
**FM2**	rs9399137-T	98	rs9402685-T	103	50	151	98	51% (in phase)	T-T and C-C
	rs9399137-T	113	rs9402685-T	124	81	156	113	72% (in phase)	T-T and C-C
	rs9399137-T	88	rs9402685-C	108	9	187	88	10% (in repulsion)	T-T and C-C
	rs9399137-T	99	rs9402685-C	91	6	184	91	7% (in repulsion)	T-T and C-C
**FM3**	rs9399137-T	193	rs9402685-T	211	185	219	193	96% (in phase)	T-T and C-C
	rs9399137-T	183	rs9402685-T	193	173	203	183	95% (in phase)	T-T and C-C
	rs9399137-T	176	rs9402685-C	125	6	295	125	5% (in repulsion)	T-T and C-C
	rs9399137-T	179	rs9402685-C	147	10	316	147	7% (in repulsion)	T-T and C-C
**FM4**	rs9399137-T	103	rs9402685-T	106	99	110	103	96% (in phase)	T-T and C-C
	rs9399137-T	128	rs9402685-T	125	118	135	125	94% (in phase)	T-T and C-C
	rs9399137-T	121	rs9402685-C	109	10	220	109	9% (in repulsion)	T-T and C-C
	rs9399137-T	117	rs9402685-C	104	7	214	104	7% (in repulsion)	T-T and C-C

Individuals FM1, 2, 3, and 4 were known to be double-heterozygous for the SNP marker pair rs9399137–rs9402685, and phase between the alleles had previously been determined through family relationships (Figure 3). Digital-array haplotyping was carried out in duplicate, with two different probe (i.e., allele) combinations each.

All tests arrived at the correct haplotype combination for all for subjects: ‘T-T’ on one chromosome, and ‘C-C’ on its sister chromosome.

The presence of 70 to 100 targets per panel appeared to be the optimum. Nevertheless, unequivocal phase data were obtained from as little as about 30 and as many as about 200 target molecules per probe present (Table 1, Tables S1 and S2).

The procedure is relatively quick, taking about 2.5 hours from DNA sample to phase result. The number of panels on each chip (array) determines the throughput. Up to 12 samples (alternatively, twelve different SNP pairs) can be haplotyped in one experiment with the smaller digital arrays, 48 samples (or SNP pairs) with the larger second-generation arrays (Figure 1).

### Confirmation of phase data in a family study

We experimentally haplotyped a pair of SNPs (rs9399137 and rs9402685) in four double-heterozygous individuals, where phase information had previously been obtained through family relationships (Figure 3). Among the test individuals, as it is typical for this locus in individuals of European and South Asian Caucasian origin, only two of the four possible haplotypes exist, i.e. T (for rs9399137)–T (for rs9402685), and C (rs9399137)–C (rs9402685). Therefore, being heterozygous, all four individuals have the same haplotype situation for this SNP pair, T - T on one chromosome, and C - C on the other (Figure 3).

Digital array haplotyping was performed four times for each person (Table 1), using two different probe combinations: allele ‘T’ for rs9399137 was paired either with allele ‘T’ or with allele ‘C’ for rs9402685. Both test combinations yielded consistent results (always showing ‘in phase’ for the first combination and ‘in repulsion’ for the second combination, Table 1) and all experiments yielded the correct haplotype make-up, identical to that found through the family study (Figure 3).

### Digital-array haplotyping of patients with sickle cell disease

We experimentally generated phase information in those 16 of the 68 patients in the study, where heterozygosity of two or three genotypes prevented the unambiguous reading of haplotypes directly from genotype data. For rs9399137–rs9402685, digital-array phasing was performed in seven patients, for rs9402685–rs11759553 in thirteen patients. All experiments were conducted at least in duplicate, which always included a reverse-phase experiment, where two alleles were paired in an alternative combination. The patient results are summarized in Table 2. Individual experiments are shown in Tables S1 and S2 and are also included in Figure 5.

**Table 2:** Summary of haplotyping results in the patients.

allele combination tested	Experiments	Targets per probe	% co-localization in phase	% co-localization in repulsion	haplotype combinations derived
rs9399137-T–rs9402685-T	n = 14	38 to 66	76% to 98%	none	‘T-T/C-C’: n = 14; ‘T-C/C-T’: none
rs9399137-T–rs9402685-C	n = 14	37 to 69	none	0% to 11%	‘T-T/C-C’: n = 14; ‘T-C/C-T’: none
rs9402685-T–rs11759553-T	n = 13	25 to 69	84% to 100%, n = 3	3% to 12%, n = 10	‘T-A/C-T’: n = 10; ‘T-T/C-A’: n = 3
rs9402685-C–rs11759553-T	n = 13	27 to 58	53% to 87%, n = 10	2% to 6%, n = 3	‘T-A/C-T’: n = 10; ‘T-T/C-A’: n = 3

The haplotype combinations detected, including those for the reverse-phase experiment, were the same for each patient. As an additional check on the accuracy of the haplotypes detected in the patients, we performed single-molecule sequencing on all seven patient samples phased for the SNP pair rs9399137–rs9402685, along with one of the family members (FM01). Phases obtained for these two markers by sequencing were identical to those from digital arrays (Table 1 and Table S1).

### Haplotype make-up of the patient population

Digital-array haplotyping resolved phase in all 16 patients with a previously unknown haplotype make-up. This now provided all information necessary to derive three-SNP haplotype signatures (rs9399137–rs9402685–rs11759553) at the HMIP-2 locus for all 68 patients (Table 3). Haplotypes directly observed in an individual occasionally differed from those identified as the most likely ones by the mathematical algorithm implemented in PHASE 2.1.1 [17] (Table 3).

**Table 3:** Presence of individual *HMIP-2* haplotypes in 68 patients of African and Afro-Caribbean ancestry.

Haplotype	Phase known through homozygosity	Inferred mathematically (most likely)	Determined experimentally	Discrepancy	Total present in patients	European population
	N =	N =	N =	N =	N = (Frequency)	Frequency
(1) T - T - A	52	14	11	3	63 (0.46)	0.71
(2) T - T - T	22	2	5	3	27 (0.20)	0.01
(3) T - C - A	15	0	3	3	18 (0.13)	<0.01
(4) T - C - T	13	9	6	3	19 (0.14)	<0.01
(5) C - T - A	0	0	0		0 (0.00)	0.00
(6) C - T - T	0	0	0		0 (0.00)	0.00
(7) C - C - A	0	1	1		1 (0.01)	<0.01
(8) C - C - T	2	6	6		8 (0.06)	0.27

The discrepancies between the mathematically inferred haplotypes and the experimentally detected ones arise from three individuals, where a haplotype combination of ‘T-T-A/T-C-T’ is the most likely one, given the individual SNP genotype data, but where a combination of ‘T-T-T/T-C-A’ is actually present.

Haplotype frequencies in British Europeans, as observed in a previous study [1], are given for comparison.

The spectrum of haplotypes occurring in the patient population is much more diverse than that previously found in Europeans. Haplotype 1 (T-T-A) is similarly prevalent in both populations. Alleles forming this haplotype are usually associated with low trait (HbF) values [1], [16]. In contrast, the other haplotype common in Europeans, haplotype 8 (C-C-T), which contains the high-trait ‘C’ allele for rs9399137, is relatively rare in the African-descended patients, and seems often admixture-derived (data not shown). Instead, in the patients the ‘intermediate’ haplotypes 2, 3, and 4 are prevalent, and it is these which would be expected to be informative for fine-mapping and functional studies.

### Three-color haplotyping

The haplotyping strategy investigated in this paper is based on establishing phase between two neighboring SNP markers with heterozygous genotypes. Haplotypes would then be expanded by ‘walking’ along the chromosome, establishing phase with the next heterozygous marker and the next after that, while phase with homozygous markers in between is dictated by the genotype already.

We have briefly explored an alternative approach, i.e. to phase three alleles at once, from three markers, using three different fluorophores. For this, the approach was identical to digital-array haplotyping described above for the pair rs9399137–rs9402685, but an additional primer pair and one ROX-labeled allele probe (Methods S1) for rs6930223 was added. This marker is 4.5 kb distal to rs9402685 and 5.2 kb distal to rs9399137. Initial results indicate that obtained co-localization scores seem to be unaffected by the presence of three colors and only marginally affected by the somewhat greater distance between markers. They were between 0 and 11.3% for alleles in repulsion, and between 54.5% and 95.9% for alleles in phase (individual data not shown).

## Discussion

We have demonstrated a simple and fast method to experimentally obtain SNP haplotype data using nanofluidic chips that allow the isolation and assaying of individual human genomic DNA template molecules. Haplotyping with such ‘Fluidigm Digital Arrays’, we investigated the *HMIP-2* disease modifier locus in patients with sickle cell disease.

Our approach represents a straightforward way to ‘add-on’ a haplotyping option to an existing genotyping method, such as the widely-used TaqMan-based allele differentiation. Haplotype data can add power to genetic mapping studies, and our method would allow researchers or clinical geneticists to utilize this additional information even when samples from family members are not available. We project that ultimately genome-wide haplotype data might be routinely used to map complex traits. In the interim, the availability of a haplotyping option for targeted loci could help to evaluate the scientific advantages of gaining this additional layer of information. Haplotypes act like individual highly informative markers harboring a series of rare alleles. The additional power gained might be critical, e.g., when mapping rare functional variation where common, less informative markers might fail to show maximum association, or in pharmacogenomic studies, where sample sizes are small and replication samples are unattainable [23]. In our own studies, genetic admixture potentially leads to allelic heterogeneity. Labeling and mapping European and African chromosomes separately in our patients might help to overcome this difficulty and make full use of the enhanced resolution [16] of African-derived chromosomes for finemapping. Additional genetic and epigenetic information, such as copy number variation (CNV [24]) or methylation imprints, might also be tied into the SNP haplotypes.

The distinct clustering of our co-localization scores (Figure 5) indicates that unequivocal phasing seems straightforward using nanofluidic arrays. Scenarios are conceivable though that would cause ambiguity: cross-contaminated, severely degraded, or over-concentrated DNA samples, as well as a too wide spacing of the SNP pair tested. For instance, the tendency of some samples to yield co-localization scores at the lower end of the in-phase cluster, such as FM2 (Table 1, Figure 5) might be due to partial strand breakage in a more degraded sample. Quality control measures implemented with our approach could include the monitoring of score clustering and the use of control samples, i.e. phase-known individuals or sample mixes with phase-known components. While we have used specifically formulated primer/probe mixes, the utilization of off-the-shelf TaqMan oligonucleotide mixes might also be possible, provided that suitable analysis algorithms can be devised.

The accidental inclusion of homozygous SNPs can lead to the detection of only one of the two expected alleles, alerting to possible genotyping errors or sample swaps.

Experimentally-derived haplotypes were found, as expected, to be not always identical to the most likely haplotype, as mathematically inferred from SNP genotypes. Where the mathematical algorithm leaves ambiguity (in our study, <90% haplotype probability), it is impossible to determine in which individuals the less probable haplotypes occur. These ‘atypical’ haplotypes would therefore be lost for functional and fine-mapping experiments. Assigning haplotypes correctly is an important aspect of our studies investigating sickle cell disease, as it would be in any undertaking comparing the haplotypes present in individuals with their phenotype or other functional data. Thus, beyond mapping studies, experimental haplotyping would be useful in experiments where exact allelic phase between polymorphisms has to be know, such as in studies of cis-regulatory effects on gene expression and other gene function [25] or when investigating adjoining and cis-interacting functional variants. A potential application in a clinical environment is the emerging field of non-invasive prenatal diagnosis [26], where a minor fraction of fetal DNA has to be identified against a majority of maternal cell-free DNA in the plasma of the mother. Here, experimental haplotyping could be used, e.g., to detect the paternal component of a recessive condition, anchoring the mutation originating from the father to alleles that are specific to the affected paternal chromosome.

It is our hope that the method described here or equivalent approaches will, in the near future, routinely add phase information to SNP genotype data, enhancing researchers' power to detect, map, and ultimately understand variation in the human genome.

## Supporting Information

Methods S1 Supporting methods information.
(0.03 MB DOC)

Table S1 Individual digital-array haplotyping experiments of patient samples with SNP marker pair rs9399137–rs9402685.
(0.02 MB XLS)

Table S2 Individual digital-array haplotyping experiments of patient samples with SNP marker pair rs9402685–rs11759553. For patient 48, no ‘T’ allele was detected for rs11759553. This conflict between the digital-array result and the original genotyping result (‘A T’ heterozygous) was subsequently resolved and the correct genotype was shown to be indeed ‘A A’ homozygous, in agreement with the digital array data.
(0.02 MB XLS)
