# Supplementary material for: Experimental Generation of SNP Haplotype Signatures in Patients with Sickle Cell Anaemia
Source: PLoS One. 2010 Sep 24;5(9):e13004. doi: 10.1371/journal.pone.0013004 (PMC2945316; doi:10.1371/journal.pone.0013004)
Supplement: Methods S1 — Supporting methods information. (0.03 MB DOC) [file pone.0013004.s001.doc]

## Supporting methods information

### Oligonucleotide sequences (5’ to 3’)

**for TaqMan assays:**

*rs9399137* (forward primer: CATCACCTTAAAAGGCGGTATTGTATG, reverse primer: GATTCCACTTTCAGAACTTATCCCAAGA, probes: AAAAACTGT***G***AATAACC–VIC, AAAAAACTGT***A***AATAACC–FAM), *rs9402685* (forward primer: TGAGATTACAGGCGCATGCAA, reverse primer: ACTGAGGCAGGTGGATTGC, probes: TTCGAGAGCA***A***CCTGA–VIC, TCGAGAGCA***G***CCTGA–FAM or –VIC),

*rs11759553* (forward primer: GGATCTACCTCTGGCTGATTGG, reverse primer: TGTTCTGCAGGGTCCTTTGG, probe: CCATAGG***T***CAGGACAT–FAM),

*rs6930223* (forward primer: CTTCCTGAAGCCTGCTGTAGA, reverse primer: GCTGACACCCACTTCAAAGAAC, probe: CTATAAGAGCGGCCCT-ROX.

**for single molecule PCR and sequencing:**

C6-F (AGACATTTTTC ATGTCATTAGATATTTTGTAC),

C6-R (TCACACCTGTAATCATGCTTTAGG).
